# Overcoming the non-kinetic activity of EGFR1 using multi-functionalized mesoporous silica nanocarrier for in vitro delivery of siRNA

**DOI:** 10.1038/s41598-022-21601-w

**Published:** 2022-10-14

**Authors:** Javad Parnian, Leila Ma’mani, Mohamad Reza Bakhtiari, Maliheh Safavi

**Affiliations:** 1 Department of Biotechnology, Iranian Research Organization for Science and Technology (IROST), P. O. Box 3353-5111, Tehran, Iran; 2 Department of Nanotechnology, Agricultural Research Education and Extension Organization (AREEO), Agricultural Biotechnology Research Institute of Iran (ABRII), P. O. Box 31535-1897, Karaj, Iran

**Keywords:** Cancer, Drug discovery

## Abstract

Triple-negative breast cancer (TNBC) does not respond to HER2-targeted and hormone-based medicines. Epidermal growth factor receptor 1 (EGFR1) is commonly overexpressed in up to 70% of TNBC cases, so targeting cancer cells via this receptor could emerge as a favored modality for TNBC therapy due to its target specificity. The development of mesoporous silica nanoparticles (MSNs) as carriers for siRNAs remains a rapidly growing area of research. For this purpose, a multi-functionalized KIT-6 containing the guanidinium ionic liquid (GuIL), PEI and PEGylated folic acid (FA-PEG) was designed. Accordingly, KIT-6 was fabricated and modified with FA-PEG and PEI polymers attached on the surface and the GuIL placed in the mesopores. Subsequent to confirming the structure of this multi-functionalized KIT-6- based nanocarrier using TEM, SEM, AFM, BET, BJH, DLS and Zeta Potential, it was investigated for uploading and transferring the anti-EGFR1 siRNAs to the MD-MBA-231 cell line. The rate of cellular uptake, cellular localization and endolysosomal escape was evaluated based on the fluorescent intensity of FAM-labelled siRNA using flowcytometry analysis and confocal laser scanning microscopy (CLSM). The 64% cellular uptake after 4 h incubation, clearly suggested the successful delivery of siRNA into the cells and, CLSM demonstrated that siRNA@[FA-PEGylated/PEI@GuIL@KIT-6] may escape endosomal entrapment after 6 h incubation. Using qPCR, quantitative evaluation of EGFR1 gene expression, a knockdown of 82% was found, which resulted in a functional change in the expression of EGFR1 targets. Co-treatment of chemotherapy drug “carboplatin” in combination with siRNA@[FA-PEGylated/PEI@GuIL@KIT-6] exhibited a remarkable cytotoxic effect in comparison to carboplatin alone.

## Introduction

Triple-negative breast cancer (TNBC), known to have a very poor prognosis, is inherently resistant to targeted chemotherapeutic drugs and is characterized as the most aggressive and lethal subtype of breast cancer. Because TNBC treatment options are so limited and ineffective, it seems essential to shift to newer generations of anticancer agents via selecting an appropriate oncogenic target^1,2^. The treatment of TNBC, metastatic lung, colon, pancreas and head & neck cancers with epidermal growth factor receptor (EGFR) inhibitors leads to multi drug resistance (MDR) in the patients. One of the basic causes of drug resistance is its non-kinetic activity, which has led to the inefficiency of kinase inhibitors administration. Despite the inactivation of the kinase activity of this receptor tyrosine kinase, the epidermal growth factor receptor 1 (EGFR1) is still functional in the spread of cancer^3^. Weihua et al. (2008) found that EGFR physically associates with sodium/glucose cotransporter (SGLT1) and stabilizes it, thereby increasing glucose uptake into cancer cells. As a result, kinase inactivation of the EGFR is not adequate to overcome TNBC. Targeting the whole mRNA of EGFR could be an efficient method to achieve both aims, i.e. deactivating kinase function and preventing kinase-independent role^4^.

As an alternative, small interfering RNA (siRNA)-based therapy can strongly silence target oncogenes expression without off-target. The overexpression of specific genes in cancer cells leads to chemo-resistance, so gene modulation and silencing through siRNA treatment seems to be the most promising strategy for re-sensitizing primarily ineffective chemotherapy in TNBC cells. However, poor cellular uptake, rapid clearance in the circulatory system and poor siRNA stability restrict the beneficial results and applications of siRNAs as successful therapeutic agents in cancers. Given these drawbacks, targeted vehicles, particularly nanomaterial-based delivery, appear to be a highly desirable and promising approach for overcoming these limitations. As previously reported, such treatments for MDR cancer cells were administered by siRNA-loaded mesoporous silica nanoparticles (MSNs)^5,6^.

MSNs have been developed as useful therapeutic tools for efficient drug and gene delivery^7–14^ due to their advantageous physicochemical properties, including extended surface area, easy surface functionality, arranged pore structure, specific pore volume, tunable pore size, biocompatibility and biodegradability^9,11^. In comparison with other drug and gene delivery systems, MSNs can not only protect numerous amounts of drug molecules via encapsulation, but also achieve controlled drug release in human physiologic conditions^15,16^. According to recent studies, the modified MSNs can simply be adsorbed by mammalian cells and display desirable biocompatibility and low toxicity^17–19^. Therefore, MSNs have become suitable platforms for the delivery of drugs and nucleic acids.

Due to the relatively small pore size of MSN to hold biomacromolecules, the therapeutic nucleic acid is adsorbed or immobilized on the surface of mesoporous silica and also encapsulated inside the engineered MSNs with large pores^20^. In MSN-based siRNA delivery studies, mostly the outer surface of these particles must be pre-operated by positively charged groups such as amino groups or polyelectrolyte-coated rich amino groups. Therefore, the positive surface charge of MSN was provided by cationic polymeric coatings, which caused the electrostatic force for adsorbing siRNA on the external MSN surface. By packing siRNA on the nanoparticles’ surface, the amino groups on the outer surface are evacuated due to electrostatic interactions. This reveals another defect caused by additional changes at the nanoparticle surface, making it difficult to bind biocompatible molecules necessary for in vivo functional purposes. As a result, siRNA loading within MSN becomes critical to exploring the area of application of these powerful carriers. To date, there have been some reports of the success of such a link between siRNA and the porous surface of MSN nanomaterials^8,21,22^.

Owing to the importance of the aforementioned obstacles in delivery of siRNA and our previous efforts, herein siRNA-based cargo was loaded on the multifunctional MSN-based nanocarrier to overcome the non-kinetic activity of EGFR1. For this purpose, the nanocarrier was modified by grafting the guanidinum ionic liquid (GuIL) groups on the inner surface and the PEGylated folic acid (PEG-FA) and polyethylene imine (PEI) on the outer surface of the pores. The prepared multifunctional MSN-based nanocarrier was applied for delivery of anti-EGFR1 siRNAs and transferred to MD-MBA-231 cell line. Besides, guanidinium based ionic liquid is a strong denaturant able to protect siRNA against nucleoside digestion^23–27^. Finally, the encapsulated siRNA into the multi-functionalized MSN-based nanocarrier was evaluated for in vitro treatment of the TNBC cell lines and analyzed its cellular uptake and quantified EGFR gene expression knockdown. Desirably, we should use a method to insert siRNA into MSN pores, in such way that the outer surface of the nanoparticle could be available for further functionalization and more simultaneous applications be made for this system, such as gene silencing, co-delivery of different nucleotides, placement of ligands for tissue-specific targeting, imaging or tracking and so on.

## Results

### Synthesis, functionalization and characterization of the nanocarrier

**Figure 1:**
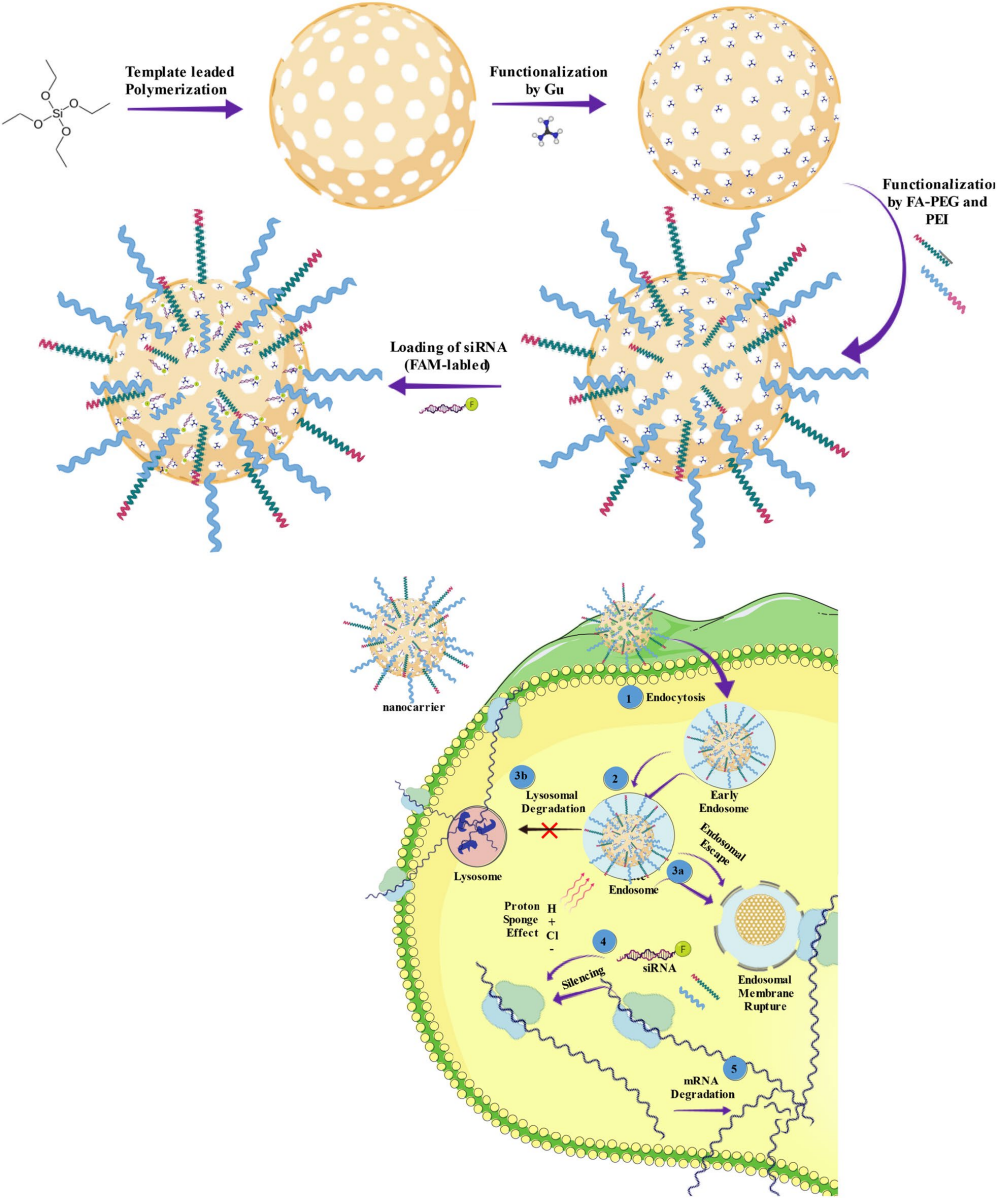
Schematic outline of the steps of modification of KIT-6 by GuIL, PEG, PEI 1and internalization of siRNA/Nanocarrier. 1. Nanocarrier enters the cell through endocytosis + /receptor-mediated endocytosis. 2. Formation of early endosome. 3a. Endosomal escape (proton sponge theory). 3b. Integration with lysosomes. 4. Incorporation of siRNA into RISC. 5. Target mRNA (EGFR1) induce cleavages and digestion.

**Figure 2:**
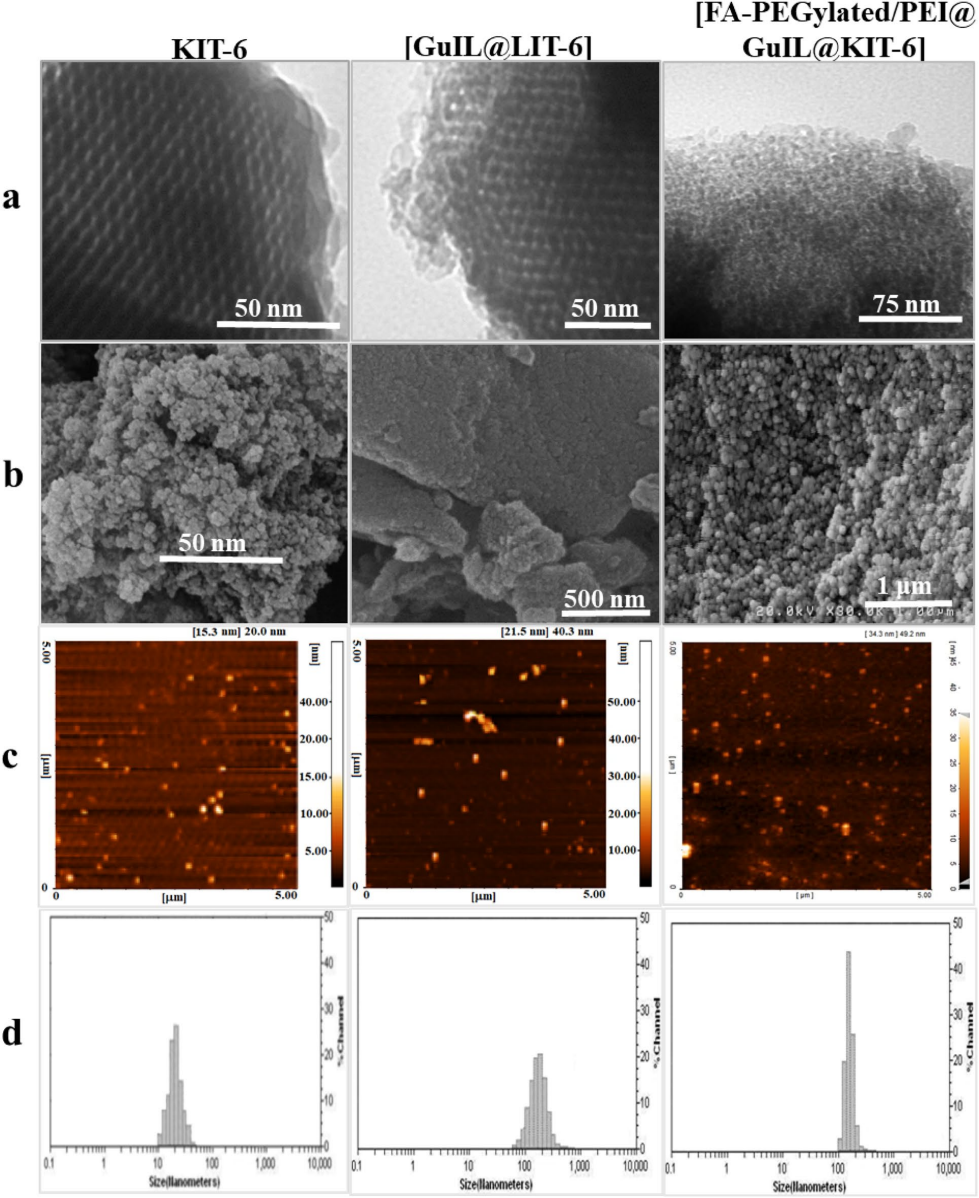

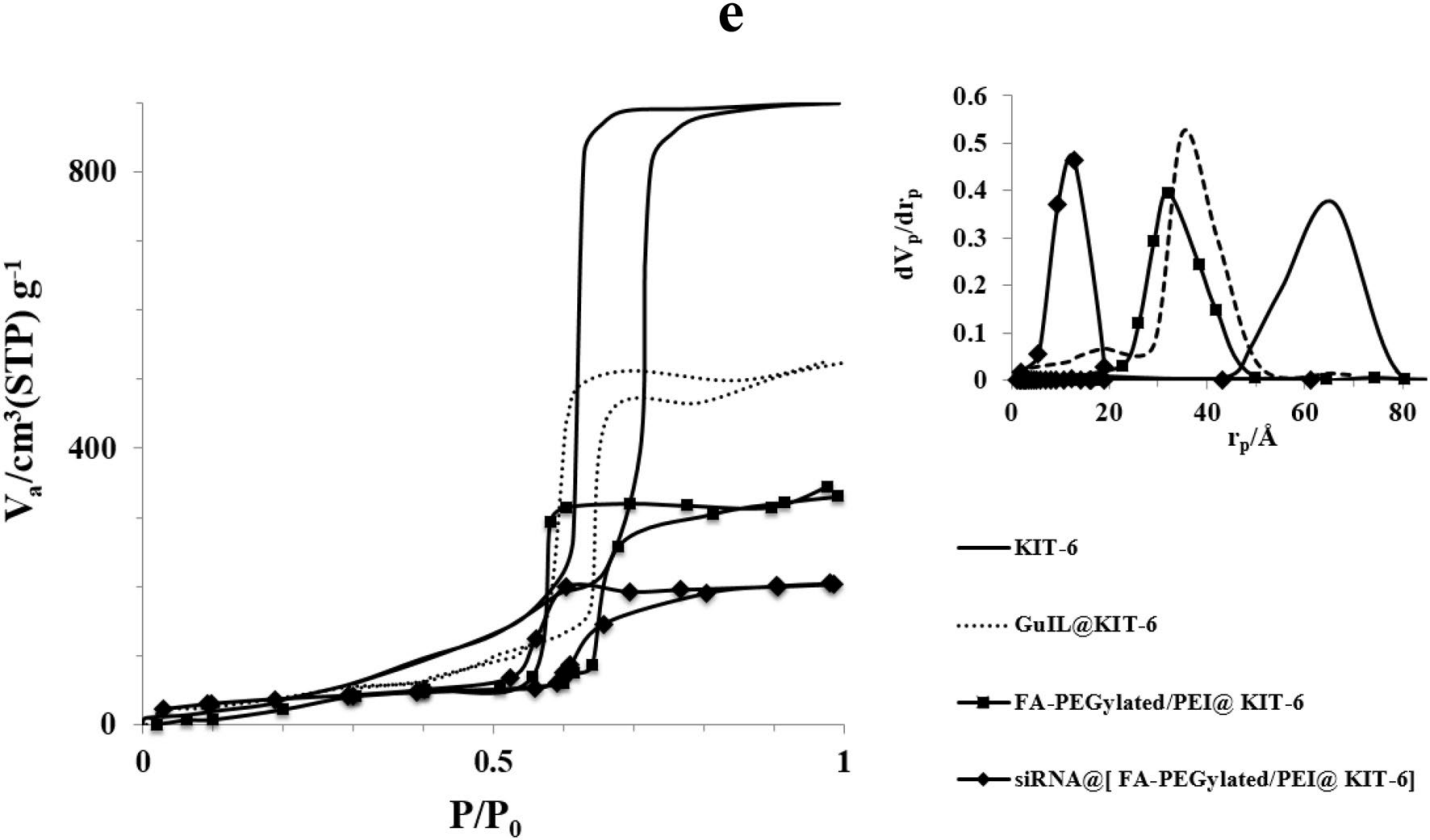
(**a**) TEM, (**b**) SEM, (**c**) AFM and (**d**) DLS images and (**e**) BET/BJH results of KIT-6, GuIL@KIT-6 and [FA-PEGylated/PEI@GuIL@KIT-6] nanoparticles.

We designed a modified KIT-6 mesoporous silica nanoparticle-based nanocarrier for efficient siRNA loading into the mesoporous. The chloropropyl functionalized KIT-6 was first prepared and then the surface of the pores decorated with GuIL functional groups. Next, the external surface of the nanocarrier was equipped. The introduction of the PEI and PEG grafted onto the MSN NPs was to increase the positive charge required for nanocarrier-cell interactions and proceed endosomal escape (proton sponge theory). Therefore, the presented nanocarrier was synthesized with a positively charged external surface. The inner surfaces of the mesoporous were also positively charged by guanidine-based ionic liquid. The diameter of the mesoporous was designed and created to be appropriate to the charge and diameter size of the siRNA molecule (~ 2 nm)^28,29^, so siRNA may enter within these positively charged mesoporous and interact with guanidine and the PEI decorated on the external surface (Fig. 1). However, guanidine may also be embedded on the external surface of the pores, as due to its anti-nuclease effects, it is ideal for protecting siRNA against nucleoside digestion. The structure of [FA-PEGylated/PEI@GuIL@KIT-6] was widely characterized by DLS, Zeta Potential, XRD, Fourier transform infrared spectroscopy (FTIR), BET/BJH, AFM, TEM and SEM (Fig. 2, Table 1, Supplementary fig. 1).

**Table 1:** The results of nitrogen adsorption/desorption for nanomaterials.

NPs	SBET (m^2^ g^−1^)	V_total_ (cm^3^ g^−1^)	Pore diameter (nm, BJH)
KIT-6	850.5	1.65	6.5
GuIL@KIT-6	525.6	0.689	3.5
FA-PEGylated/PEI@ KIT-6	330	0.624	3.1
siRNA@[FA-PEGylated/PEI@ KIT-6]	204	0.219	1.2

XRD analysis confirmed the meso-porosity with a symmetry of the space group *Ia3d* for [FA-PEGylated/PEI@GuIL@KIT-6] and siRNA@[FA-PEGylated/PEI@GuIL@KIT-6] samples. The diffraction peaks including [211], [220] and [332] reflections were seen at 2Ɵ = 0.78°, 0.98° and 1.65°, respectively. This result confirmed the retention of the mesoporous nature of the nanocarrier during the loading process. TEM and SEM were applied to realize the morphology and structural order of the MSN-based nanocarrier (Fig. 2). According to TEM images, the synthesized nanocarrier before and after modification possesses an ordered arrangement of honeycomb-like network with uniform pore size. The mean pore size of the [FA-PEGylated/PEI@GuIL@KIT-6], calculated by the BJH, was about 3.5 nm. As seen in Fig. 2, the average particle size of the [FA-PEGylated/PEI@GuIL@KIT-6] generated was 75 nm with a size variation of ± 7 nm. According to previous research, the ideal size of nanoparticles for efficient gene delivery is less than 75 nm^30,31^. As a result, the [FA-PEGylated/PEI@GuIL@KIT-6] nanocarrier had the suitable size for effective gene transport. AFM image has represented a uniform 3D topography image for the [FA-PEGylated/PEI@GuIL@KIT-6] nanocarrier.

A zeta potential study was carried out to assess the MSN-based nanocarrier stability surface charge, and its potential for efficient interaction with siRNA as a cargo. The zeta potential results displayed that the surface charges were attributed to the existence of GuIL, FA-PEG, PEI and siRNA in the structure of samples, and the KIT-6, FA-PEGylated@GuIL@KIT-6, [FA-PEGylated/PEI@GuIL@KIT-6] and siRNA@[FA-PEGylated/PEI@GuIL@KIT-6] complexes had charges about of − 25, − 3, 39 and 18 mV, respectively. Positive and negative zeta potentials can be utilized to determine whether PEI or siRNA prevails at the MSN complex surface and pores, since PEI is positively and siRNA negatively charged. The siRNA@[FA-PEGylated/PEI@GuIL@KIT-6] complex was created by electrostatic interaction between the positively charged PEI and GuIL groups with the negatively charged siRNA-cargo. This interaction led to a decrease of the zeta potential from 39.75 mV in [FA-PEGylated/PEI@GuIL@KIT-6] to 18 mV in siRNA@[FA-PEGylated/PEI@GuIL@KIT-6]. These findings are consistent with those previously published^32,33^. The positively charged [FA-PEGylated/PEI@GuIL@KIT-6] due to PEI and guanidinium ionic liquid groups allowed the siRNA to bind firmly to its surface. As a result, PEI and GuIL preserve siRNA while also allowed for effective delivery of siRNA to cancer cells.

The presence of functional groups on functionalized MSN was confirmed by FTIR analysis. The stretching vibration of the -OH group originating from the hydroxyl group of nanoparticles and water molecule exhibits a wide absorption peak from 2972 to 3339 cm^−1^ in the FTIR spectra of KIT-6, [GuIL@KIT-6], [FA-PEGylated/PEI@GuIL@KIT-6] and siRNA@[FA-PEGylated/PEI@GuIL@KIT-6]^34,35^. The wide absorption peaks at about 3430 and 924 cm^−1^ in the FTIR spectrum of MSN sample might be attributed to the stretching and bending vibrations of hydroxyl (O–H) bonds existing on the surface Si–OH and the absorbed water molecules, respectively^36^. The stretching vibrations of the Si–O–Si bonds on the Si-skeleton might be responsible for the strong peaks at 948–1085 cm^−1^. The band at about 2910 cm^−1^ in the spectra of PEG_600_-MSN might be ascribed to stretching vibration of aliphatic C–H bonds of the PEG chain. In addition, the apparent rise in the band’s intensity around 3430 cm^−1^ might be attributed to the stretching vibration of the PEG chain’s O–H bond. These analyses concluded that PEG molecule successfully changed MSN frameworks. The bands at 2954, 2868 and 1020 cm^−1^ in the spectra of [FA-PEGylated/PEI@GuIL@KIT-6] could be referred to the vibrations of the C-H bonds, while the other band at 1465 cm^−1^, to the stretching vibration of the C–N bonds present in the grafted organic groups (such as Gu)^37^. The CO–N bond is represented by the band at 1540 cm^−1^, whereas the exterior of the plane bending vibrations of = C–H and -CH_2_ are represented by at 759 cm^−1^. PEI revealed absorption peaks related to C–N stretching and bending vibrations at 1355 cm^−1^ and 764 cm^−1^, respectively. In PEI, the aromatic ether C–O–C peak occurs between 1050 and 1150 cm^−1^. Due to surface coating, the aromatic ether C–O 1286 cm^−1^ was displaced to 1394 cm^−1^ in the FTIR spectrum. As a result, we confirmed that the PEI layer was coated on the MSNs’ surface.

Specific surface area was calculated using the multiple-point Brunauer–Emmett–Teller (BET) technique. The pore size distribution was calculated by the Barrett–Joyner–Halenda (BJH) method, which was based on the adsorption branch. Based on the results of the N_2_ adsorption–desorption isotherm experiment, the KIT-6 has a significant amount of both pore volume and surface area. Table 1 displays the findings, which show that after surface functionalization, surface area, pore volume, and pore size all decreased. It also revealed that the isotherms were of the type IV according to the IUPAC classification, and that they possessed a distinct H1 hysteresis cycle at relative pressures ranging from 0.5 to 0.7. This is a characteristic of pores that are similar to channels. The calcined KIT-6 has an average BJH pore diameter of 6.5 nm and a total pore volume of 1.65 cm^3^ g^−1^, and BET surface area of 850.5 m^2^g^−1^. The [FA-PEGylated/PEI@GuIL@KIT-6] and siRNA@[FA-PEGylated/PEI@GuIL@KIT-6] samples had less surface area, pore volume and pore size than the unaltered KIT-6.

These textural findings demonstrated that the grafted groups are found both within and outside of the mesoporous materials, not just on the surface. Also, following the binding of GuIL and siRNA, the pore volume and pore size showed a significant decrease, which can be attributed to the loading on both the internal and external surfaces of mesoporous.

### Gel electrophoresis retardation assay

**Figure 3:**
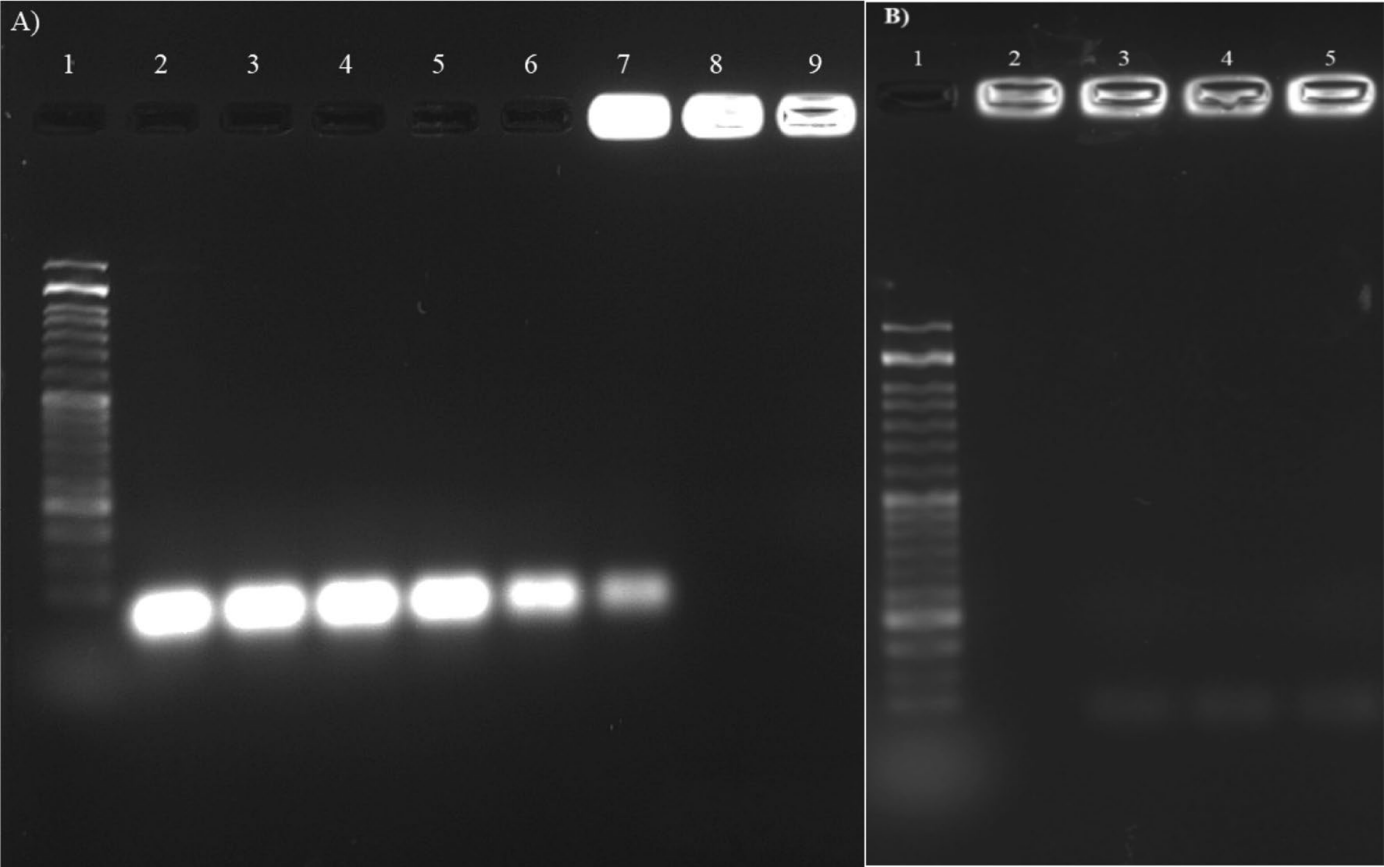
Gel electrophoresis retardation assay of (**A**) siRNA@[FA-PEGylated/PEI@GuIL@KIT-6] complex with different proportions (Lane 1): Marker (50 bp), (Lane 2): siRNA, (Lane 3): (1:1), (Lane 4): (1:2), (Lane 5): (1:6), (Lane 6): (1:12), (Lane 7): (1:25), (Lane 8): (1:35), (Lane 9): (1:50). All proportions are according to (siRNA: [FA-PEGylated/PEI@GuIL@KIT-6]). (**B**) siRNA@[FA-PEGylated/PEI@GuIL@KIT-6] with various pore sizes. (Lane 1): Ladder (50 bp), (Lane 2): Nanocarrier MSNp = 2.5, (Lane 3): Nanocarrier MSNp = 3.5, (Lane 4): Nanocarrier MSNp = 6.5 and (Lane 5): Nanocarrier MSNp = 12.

The ability of MSN-based nanocarrier to bind to nucleic acid is one of the requirements of gene delivery systems in gene therapy. In order to investigate the binding of the complex components to siRNA and the formation of the final complex, a gel electrophoresis retardation assay was used. In this method, first the final complex (siRNA@[FA-PEGylated/PEI@GuIL@KIT-6]) was loaded on a 2.5% agarose gel in different ratios of MSN to a constant amount of siRNA (2.1 µM). As shown in Fig. 3, at higher ratios, the positive charge of the polymer overcame the negative charge of siRNA and the complex remained motionless in the well and did not move towards the positive pole due to the positive or neutral net charge. While in lower proportions it could not prevent the complete delay of nucleic acid movement on the gel. This result indicates that the PEI and GuIL groups bind well to siRNA. It was assumed with increasing the ratio of the [FA-PEGylated/PEI@GuIL@KIT-6] in the complex, the net charge of the whole complex (siRNA@[FA-PEGylated/PEI@GuIL@KIT-6]) becomes neutral (or positive) and does not move in the gel. As shown in Fig. 3, while the ratio of MSN was not sufficient to provide enough positive charge to neutralize the negative charge of siRNA, siRNA moves toward the positive electrode in the gel. However, at a proportion of 1:35 (or higher ratios) of siRNA to [FA-PEGylated/PEI@GuIL@KIT-6], the whole complex has a neutral net charge, which clearly shows that the final complex is well formed.

In order to investigate the effect of pore diameter on the amount of loaded siRNA, different nanoparticles with different average pore diameters were synthesized and complexed with siRNA (Fig. 3B).

Mesoporous nanoparticles with porosities of 2.5, 3.5, 6.5 and 12, (called MSN_p=2.5_, MSN_p=3.5_, MSN_p=6.5_ and MSN_p=12_, respectively) showed different mobility. If any complexed nanoparticle with siRNA remains in the well, it is due to neutralization of the negative charge of siRNA by positive charge of the nanoparticle. As shown in Fig. 3B the net charge in siRNA@MSN with smaller and larger pore size could not become neutral (or positive), and thus moved in the gel.

### Loading efficiency

**Figure 4:**
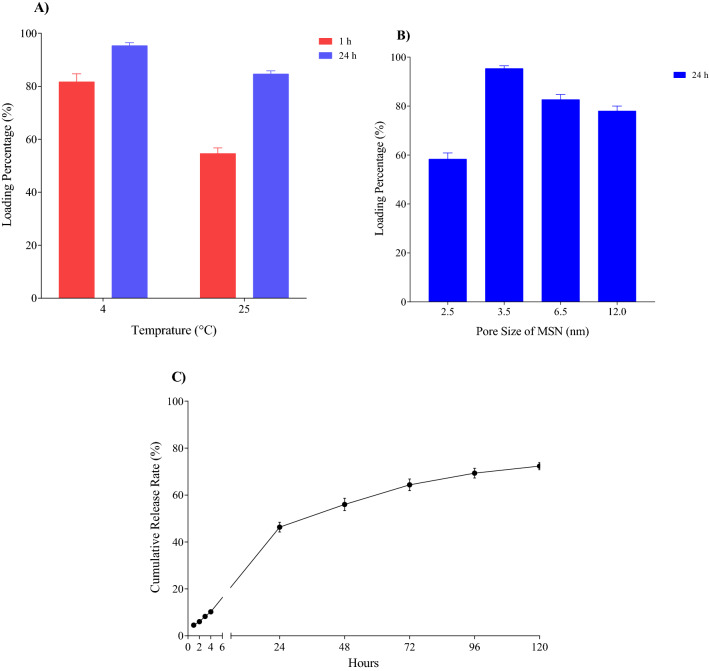
siRNA loading and releasing profile. (**A**) Loading profile of siRNA on [FA-PEGylated/PEI@GuIL@KIT-6] nanocarrier in 1 and 24 h incubation time at 4 and 25 °C. The best loading results of final complex achieved to 96% of loading in 24 h of incubation at 4 °C, with ratio of 1:35 of siRNA to [FA-PEGylated/PEI@GuIL@KIT-6] equivalent to 2.1 µM siRNA in combination with 48.3 µg of [FA-PEGylated/PEI@GuIL@KIT-6]. (**B**) Loading profile of siRNA on [FA-PEGylated/PEI@GuIL@KIT-6] nanocarrier with various pore sizes in 24 h incubation time at 4 °C (optimum condition). (**C**) The siRNA release from the [FA-PEGylated/PEI@GuIL@KIT-6] at 37 °C. Data are given as the means ± S.D. (n = 3).

In order to optimize the loading efficiency of siRNA for later applications in cell experiments, the reaction temperature and incubation time were examined by UV absorption measurements. To optimize the siRNA loading in [FA-PEGylated/PEI@GuIL@KIT-6] nanocarrier, the effect of 1:35 ratio of siRNA/MSN at 4 and 25 °C at 2 times (1 and 24 h) was evaluated. After incubation of [FA-PEGylated/PEI@GuIL@KIT-6] with siRNA, the concentration of unbounded siRNA was measured. In order to determine the optimal method of preparation of the siRNA@[FA-PEGylated/PEI@GuIL@KIT-6], different modes were investigated by considering time and temperature variables. The best result was obtained when the incubation time of nucleic acid with nanoparticles was 24 h at a temperature of 4 °C. Under these conditions, 96% of the initial nucleic acid content interacts with the nanoparticles (Fig. 4A), and this was achieved by centrifuging the mixture and reading the supernatant with the nanodrop.

In addition, to loading efficiency of siRNA in KIT-6-based nanoporous with different pore sizes was evaluated by observing the absorbance of siRNA at 260 nm in the supernatant of the centrifuged complexes. To this, MSN nanoparticles with a pore size including MSN_p=2.5_, MSN_p=3.5_, MSN_p=6.5_ and MSN_p=12_ were investigated. The amount of the loading for MSN_p=2.5_, MSN_p=3.5_, MSN_p=6.5_ and MSN_p=12_ were determined equal to 59%, 96%, 83% and 78%, respectively. It can be claimed that the results of the assays are consistent with the theoretical calculations to determine the diameter of the pores. In the nanocarriers with a larger pore size as well as smaller pores, loaded siRNA was significantly lower than the nanocarrier presented in the research (~ 96% with ~ 3.5 nm pore size)^22,38^ (Fig. 4B).

### siRNA release from MSN

The in vitro siRNA release profiles of the [FA-PEGylated/PEI@GuIL@KIT-6] was calculated by comparing the proportion of siRNA released to the initial amount of siRNA loaded into the MSNs. The release profile of siRNA-was likewise characterized by an initial release and a lag-time continuing release, as seen in Fig. 4C, the quantity of cumulative siRNA released from siRNA-MSN was around 74% after 120 h at 37 °C. These findings confirmed that a constructed nanocarrier has a slow release behavior. The amount of siRNA released in the supernatant was evaluated at different time intervals. The procedure was performed non-continuously (intermittently), in which the supernatant was discarded completely after measuring nucleic acid concentration, and the same amount of TE buffer was replaced.

### RNase A protection assays

Agarose gel electrophoresis analysis was used to confirm that [FA-PEGylated/PEI@GuIL@KIT-6] could protect siRNA from RNase enzymatic cleavage.

**Figure 5:**
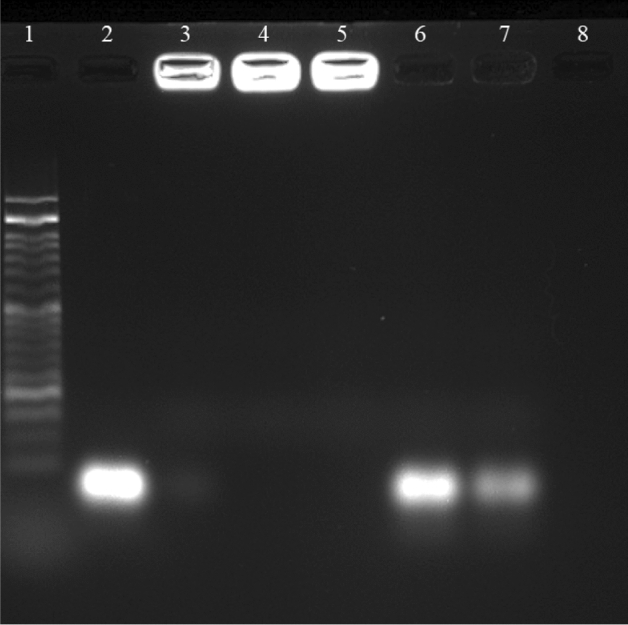
RNase protection assay for the protective effect of [FA-PEGylated/PEI@GuIL@KIT-6] on siRNA. Lane 1**:** DNA Ladder (50 bp); Lane 2**:** naked siRNA; Lane 3**:** siRNA@[FA-PEGylated/PEI@GuIL@KIT-6] (1:35); Lane 4**:** siRNA@[FA-PEGylated/PEI@GuIL@KIT-6] + RNase, 2 h; Lane 5**:** siRNA@[FA-PEGylated/PEI@GuIL@KIT-6] + RNase, 6 h; Lane 6**:** siRNA@[FA-PEGylated/PEI@GuIL@KIT-6] + RNase, 2 h + then Heparin: Lane 7**:** siRNA@[FA-PEGylated/PEI@GuIL@KIT-6] + RNase, 6 h + then Heparin; Lane 8**:** naked siRNA incubated with RNase.

To study the protective effect of siRNA by MSN (Fig. 5), the siRNA@[FA-PEGylated/PEI@GuIL@KIT-6] (siRNA 1:35 MSN) was treated with RNase A (Lanes 4 and 5), or with RNase A followed by heparin (Lanes 6 and 7). Lane 2 shows gel migration of naked siRNA. In lane 8, naked siRNA was degraded after RNase A treatment. The siRNA bound to [FA-PEGylated/PEI@GuIL@KIT-6] showed no sign of degradation after 2 and 6-h incubation time and was retained in the gel wells (Lane 4 and 5). When siRNA@[FA-PEGylated/PEI@GuIL@KIT-6] were treated by heparin after 2 and 6 h incubation by RNase, abundant release of siRNA was observed in Lane 6 and 7.

### Biocompatibility and cytotoxicity studies

**Figure 6:**
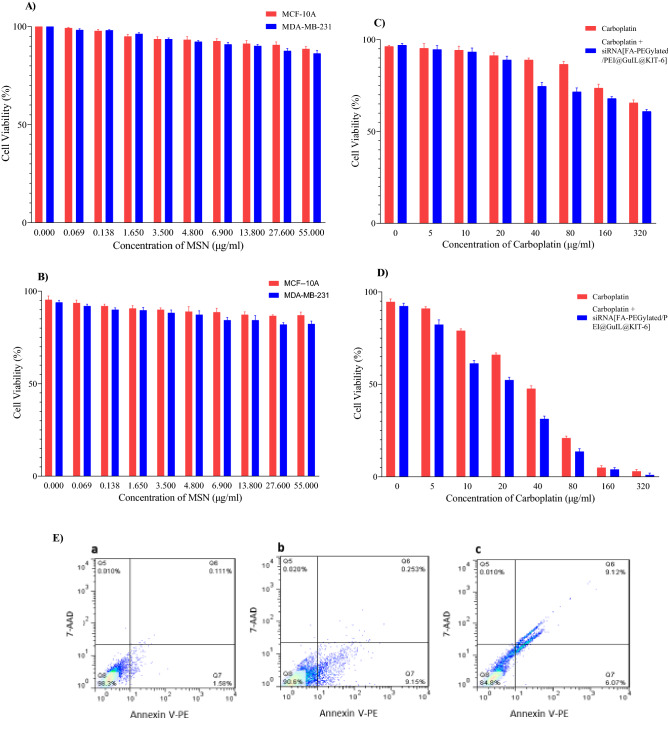
In vitro biocompatibility, cytotoxicity and apoptosis inducing activity of [FA-PEGylated/PEI@ GuIL@KIT-6], siRNA@[FA-PEGylated/PEI@GuIL@KIT-6] and carboplatin. (**A)** and **(B**) MDA-MB-231 and MCF-10A cell viability after 24 and 48 h treatment with different concentrations of [FA-PEGylated/PEI@GuIL@KIT-6], respectively. (**C)** and **(D**) MDA-MB-231 cell viability under treatment of different concentrations of carboplatin and the cocktail of various concentrations of carboplatin plus consistent concentration of siRNA@[FA-PEGylated/PEI@GuIL@KIT-6] after 24 and 48 h, respectively. (The concentration of siRNA was consistent and equal to 2.1 µM). (**E**) Flow cytometry analysis of MDA-MB-231 cells treated with carboplatin and siRNA@[FA-PEGylated/PEI@GuIL@KIT-6]. Cells were stained with Annexin V-PE/7-AAD and quantitated by flow cytometry after 24 h of incubation. The cells treated (**a**) with PBS (negative control) (**b**) with IC_50_ value of carboplatine and (**c**) cocktail of IC_50_ value of carboplatin and siRNA@[FA-PEGylated/PEI@GuIL@KIT-6] equal to 2.1 µM siRNA. Data are given as the means ± SD. (n = 3).

To be used as a siRNA delivery nanocarrier, the [FA-PEGylated/PEI@GuIL@KIT-6] was expected to have low cytotoxicity. In this regard, [FA-PEGylated/PEI@GuIL@KIT-6] was evaluated to determine whether the complex could affect cell proliferation, or pose any cytotoxicity. The MDA-MB-231 and MCF-10A cells were treated with an increasing amount of MSN for 24 and 48 h. According to the results of Fig. 6A,B the [FA-PEGylated/PEI@GuIL@KIT-6] shows no significant cytotoxicity at tested concentrations against both MDA-MB-231 and MCF-10A, at 24 and 48 h, demonstrating the compound’s excellent cytocompatibility.

Carboplatin, is currently one of the first or second line cytotoxic agents of adjuvant chemotherapy for breast cancer patients. MTT results (Fig. 6C,D) show a significant decrease in MDA-MB-231 cell viability after treatment with carboplatin plus siRNA@[FA-PEGylated/PEI@GuIL@KIT-6]. Co-treatment of carboplatin and nanoparticles containing siRNA exhibited a remarkable cytotoxic effect with IC_50_ equal to 19.19 ± 0.87 µg/mL, whereas carboplatin alone decreased the cell viability to a smaller extent (carboplatin IC_50_ = 31.69 ± 0.64 µg/mL) after 48 h incubation.

### Apoptosis induction by carboplatin/siRNA

Annexin V-PE/7-AAD kit (catalog number: E-CK-A216 Elabscience) was used to quantify cell apoptosis in order to further validate the synergistic effect of siRNA and carboplatin. Phosphatidylserine, a membrane phospholipid that is exposed to the exterior cellular environment in apoptotic cells, has a high affinity for annexin V. In order to assess the apoptosis induction of carboplatin and siRNA@[FA-PEGylated/PEI@GuIL@KIT-6] in MDA-MB-231 cells, the IC_50_ value of carboplatin 31.69 ± 0.64 µg/mL and nanoparticle containing 2.1 µM siRNA were used. The apoptotic rate of MDA-MB-231 cells significantly increased 24 h after transfection with the siRNA@[FA-PEGylated/PEI@GuIL@KIT-6]/carboplatin cocktail (Fig. 6E). In cells treated only with carboplatin, the percentage of annexin V-positive cells were 9.15 (early apoptosis). On the contrary, incubating siRNA@[FA-PEGylated/PEI@GuIL@KIT-6]/carboplatin cocktail resulted in higher cell apoptosis up to 15%, which is significantly higher than carboplatin alone. Consequently, it was concluded that carboplatin and siRNA against the human EGFR1 had a synergistic impact on in vitro human MDA-MB-231 cell death.

### Cellular uptake, localization and endolysosomes escape

**Figure 7:**
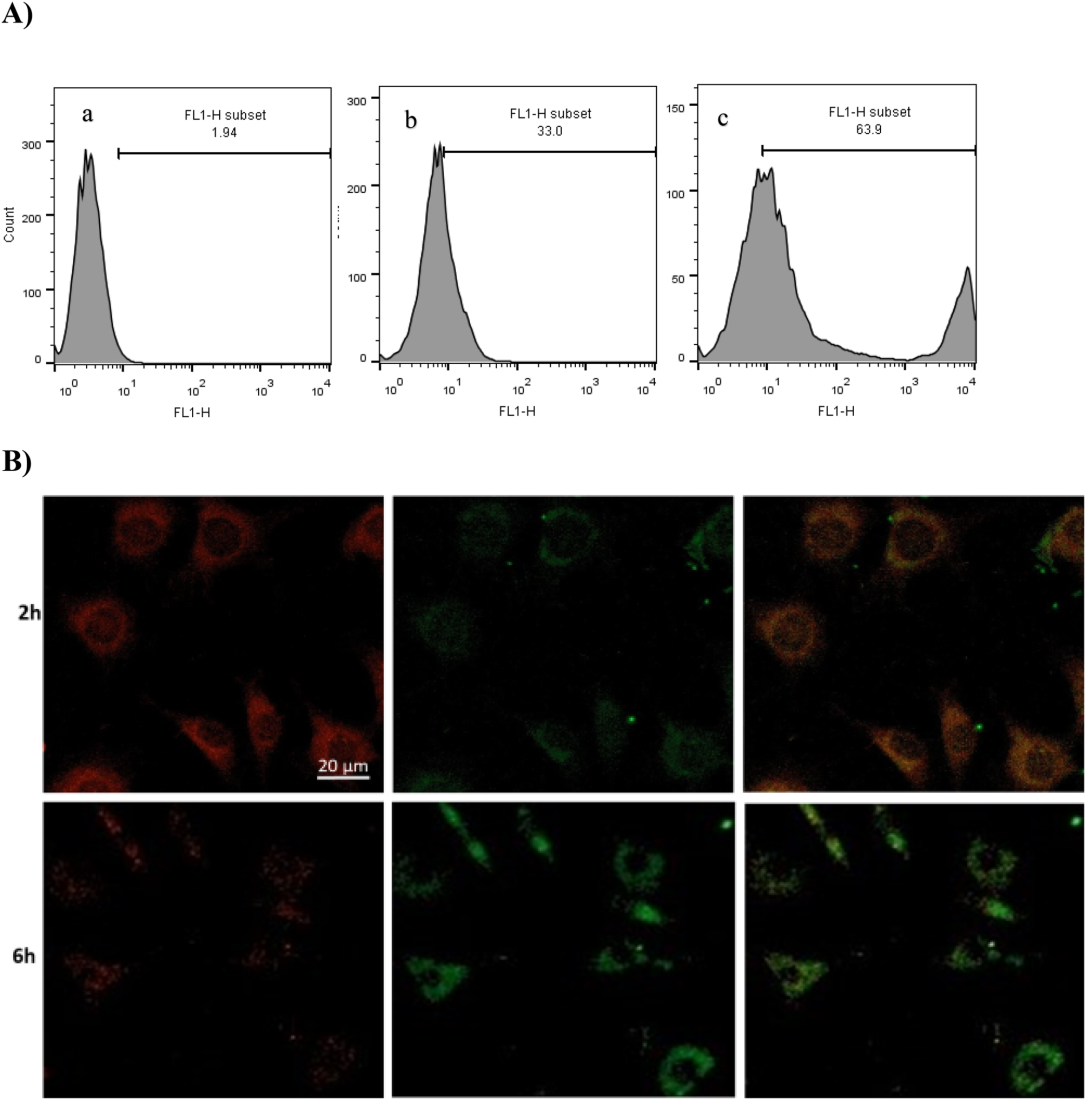
Uptake, localization and endosomal escape of [FA-PEGylated/PEI@GuIL@KIT-6] containing FAM-labeled siRNA. (**A**) Flowcytometry analysis of MDA-MB-231 cells treated with siRNA@[FA-PEGylated/PEI@GuIL@KIT-6]. (**a**) cells treated with PBS as negative control, (**b**) cells treated with siRNA@[FA-PEGylated/PEI@GuIL@KIT-6] after 2 h, (**c**) cells treated with siRNA@[FA-PEGylated/PEI@GuIL@KIT-6] after 4 h. (**B**) Confocal microscopy images of MDA-MB-231 cells treated with siRNA@[FA- PEGylated/PEI@GuIL @KIT-6] for 2 and 6 h. The NPs, and endosome were labeled with FAM (green) and LysoTracker (red), respectively.

It should be noted that effective cell internalization of MSN as a nanocarrier plays an important role in the delivery of loaded siRNA into the cancer cells. After 2 and 4 h incubations of cancer MDA-MB-231 cells with FAM labeled siRNA as reporter molecule loaded in [FA-PEGylated/PEI@GuIL@KIT-6], the cells were collected, harvested and prepared for flow cytometry analysis. According to the results, the cell adsorption rate of (siRNA@[FA-PEGylated/PEI@GuIL@KIT-6]) reached 63.9% (Fig. 7A) within 4 h of incubation time. After that, we investigated the intracellular distribution of siRNA molecules in order to further confirm that the positive surface charge provided by PEI and guanidine enabled the endocytosis and escape of entrapped siRNA from endolysosomes. The intracellular presence of siRNA@[FA-PEGylated/PEI@GuIL@KIT-6] was observed by CLSM. Treatment of the MDA-MB-231 cells with FAM-labeled siRNA@[FA-PEGylated/PEI@GuIL@KIT-6] and deep red LysoTracker exhibited green and red fluorescence co-localization, resulting in an orange signal in the merged photographs. As shown in Fig. 7B, stronger orange fluorescence (the overlap of red and green) indicates that the siRNA@[FA-PEGylated/PEI@GuIL@KIT-6] were uptake by the MDA-MB-231 cells and encapsulated into the endolysosomes after 2 h incubation.

The escape from the endosomal-lysosomal pathway following successful internalization is one of the most important processes for efficient intracellular siRNA delivery. It is critical for the carrier to promote escape from these pathways in order for siRNA to be available within the cytosol to initiate RNAi. After incubation for 6 h, the red fluorescence was found to decrease. This charge reversal of siRNA@[FA-PEGylated/PEI@GuIL@KIT-6] is thought to be responsible for their endosomal rupture and subsequent escape from the endolysosomes. As demonstrated in Fig. 7B, the red fluorescent signal of endolysosomes decreased and the green fluorescent indication of siRNA rose after 6 h of incubation.

### EGFR1 gene knockdown

**Figure 8:**
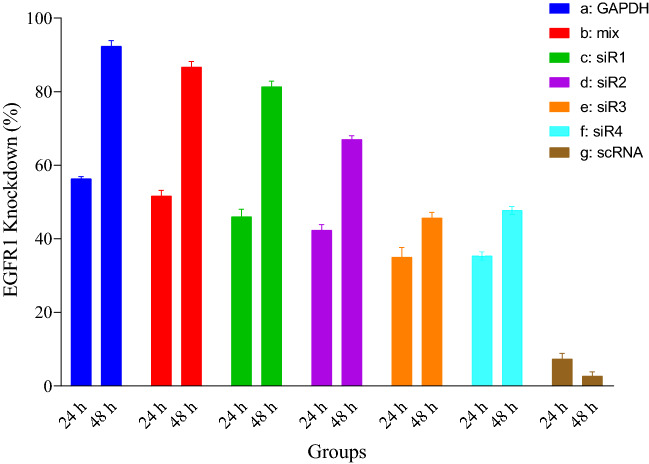
Quantitative PCR of MDA-MB-231 cells after 24 and 48 h after transfection of siRNA@[FA-PEGylated/PEI@GuIL@KIT-6]. (a) RT-qPCR analysis for positive control (GAPDH) shows about 92%, (b) for cocktail (mix) siRNAs shows about 87%, (c) for siR1 about 81%, (d) for siR2 about 67%, (e) for siR3 about 45% and (f) for siR4 about 48% knockdown of baseline expression of EGFR1 gene. (g) For scrambled RNA (scRNA) no significant change in expression level was observed.

To investigate the knockdown amount of EGFR1 at transcriptional levels, performing RT-qPCR was considered. Hence, the EGFR1 transcription levels from the examined cells were measured during 48 h of post-transfection and compared to that of the untreated cells. Normalized to the endogenous levels of the transcripts in non-transfected cells, the expression levels of the EGFR1 were evaluated. Based on the results obtained, all the statistical triplicate showed a significant knockdown of EGFR1 mRNA, 52% at 24 h and 87% at 48 h after transfection (Fig. 8).

## Discussion

In the present study, a multifunctionalized KIT-6-based nanocarrier was synthesized. The potential of the engineered mesopores was used to transfer the siRNA. We reached the goal of the study by functionalizing the nanoparticle with PEI to provide additional positive charge for subsequent purposes, including effective transfection and endosomal escape. PEG was also used for biocompatibility, and guanidinium ionic liquid was used for interaction with siRNA.

MSNs provide some benefits due to their unique mesoporous structure, which may enable therapeutically acceptable nanoformulations for treatment. MSNs have a particle size that is readily tunable, a uniform pore size, a large pore volume, a larger surface area and a simple mesoporous or hollow structure. MSNs are ideal nanoplatforms for the development of smart drug delivery systems in biomedical applications^39^.

MSNs have a high drug loading capacity and drug release patterns that are stimuli-responsive. They have good biocompatibility in vitro and in vivo. MSNs are very straightforward to functionalize, and their magnetic, fluorescent and photothermal capabilities enable nanotheranostics to perform simultaneous bioimaging and drug administration^40^. siRNA is highly negatively charged due to its phosphate backbone, so in order to bind negatively charged siRNA to MSN for improving the cellular uptake, silica nanoparticles are typically modified with positively charged organic compounds, such as PEI or poly-l-lysine (PLL)^41–43^. Consequently, in this study, cationic polymers such as PEI and GuIL moieties were incorporated to make the [FA-PEGylated/PEI@GuIL@KIT-6] positively charged for binding to siRNA electrostatically. Additionally, it has been shown the “proton sponge effect” of PEI and GuIL promotes nanoparticles’ endosomal escape that relies on an increase in H^+^ concentrations during hydrolysis in the endosomes^42,44,45^. Cationic PEI results in an increase in potential of membrane and counter-ions influx, which leads to osmotic swelling and bursting of the endosome, finally resulting in the release of siRNA to the cytosol before hydrolysis^46,47^.

The siRNA release behavior of [FA-PEGylated/PEI@GuIL@KIT-6] was studied in TE buffer at different time intervals. Probably, the released phase in the release assay was primarily caused by the progressive erosion or degradation of the MSNs matrix and the siRNA diffusion through the MSNs matrix^48,49^. The observed gradual release suggests this compound as a desirable drug carrier for our goal.

In our experiments, [FA-PEGylated/PEI@GuIL@KIT-6] was found to exhibit a notable protection efficacy after 2 and 6 h incubation with RNase. The diameter of siRNA is 2–3 nm^28,29^. As our nanoparticles have an average pore width of 3.5 nm, siRNA might potentially enter MSN pores. Since no particular siRNA orientation is necessary, and since the large positive charge of PEI can protect it against nuclease activity, some portion of the bound siRNA is more likely to bond to the outer surface.

The pore size of the nanocarriers in this study was designed and synthesized based on the diameter of siRNA (~ 2.6 nm)^28,29^. In pores smaller than this diameter, siRNA cannot enter the pore due to steric hindrance, so practically the pores lose their effectiveness and only the surface of the nanocarrier remains as a substrate for the interaction of guanidine and siRNA, which reduces the amount of siRNA in the nanocarrier. In pores larger than the diameter of siRNA, due to the large pore size, enough space is created for the reaction of other factors such as PEI and PEG, so siRNA, which will be the last interacting molecule, will face the steric hindrance of PEG and PEI to enter the pores. The results of the experiments show that the best pore diameter is slightly larger than the diameter of siRNA, so that there is space for siRNA to enter. According to our results nanoparticle with a pore size of 2.5 nm has the lowest amount of siRNA absorption equal to 59%. Nanoparticle with a pore size of 3.5 nm has the highest amount of siRNA absorption equal to 96%. Nanoparticles with pore sizes of 6.5 and 12 nm have absorbed 83 and 78% of siRNA, respectively (Fig. 4). According to the gel retardation experiment, for the tested nanoparticles with different pore sizes (2.4, 6.5 and 12 nm), we require a higher proportion of siRNA/MSN in order to neutralize the negative charge of siRNA by the positive charge of the nanoparticle (data not shown).

PEI-MSNs demonstrated much reduced cytotoxicity in cells when compared to other nonviral transfection vehicles, such as Lipofectamine 2000 and PEI/siRNA complexes^9^, confirming that PEI-MSNs offer a better approach for siRNA delivery^50,51^. The results of cytotoxicity test on cancer and normal breast cells exhibited no significant cytotoxicity of MSN after 24 and 48 h treatment. Cocktail therapy is one of the most effective methods in the treatment of various cancers. The combination of different chemical drugs or the combination of a drug with siRNA can be led by considering the mechanism of action of each component and using appropriate drug targets to create favorable conditions in cancer cells at the level of research and also using treatment methods.

By using carboplatin, DNA damage is induced, which impairs cell replication and transcription, resulting in cell death^52^. On the other hand, by using siRNA against the EGFR1 gene, the growth hormone receptor protein synthesis is silenced in the cell, resulting in a gradual decrease in the number of receptors on the cell surface. Based on the obtained results, it is reasonable to conclude that carboplatin did not operate properly in the first 24 h of the cell viability test; the explanation for this may be found in the mechanism of carboplatin action. Because in the mentioned test, a fixed number of cells are seeded, which by inhibiting their proliferation in the first 24 h after sticking (something equivalent to half the rate of duplication of MDA-MB-231 cells in ~ 42 h)^53^ cannot be expected to have a significant effect. However, carboplatin has a greater effect in 48 h, which is nearly similar to the rate of cell duplication (~ 42 h).

When siRNA is used along with carboplatin, in 48 h, there are less growth hormone receptor proteins on the cell surface and carboplatin have inhibited cell proliferation to a great extent. The effect of carboplatin and siRNA together has a more favorable effect on the survival of cells, which is caused by their growth and proliferation.

The double staining flow cytometry analysis revealed early apoptosis induced by carboplatin and also, early and late apoptosis induction in MDA-MB-231 cells treated by cocktail of the carboplatin and siRNA@[FA-PEGylated/PEI@GuIL@KIT-6] after 24 h treatment.

In comparison with other studies, the amount of transfection is desirable according to MDA-MB-231 cell line^54,55^. It should be noted that transfection reagents have various effects on different cell lines. In the case of MDA-MB231 cells, which are among the hard-to-transfect cells, this rate is reported to be up to 55% for industrial transfection reagents. According to flowcytometry analysis, MDA-MB-231 cells treated with siRNA@[FA-PEGylated/PEI@GuIL@KIT-6] for 4 h could absorb 63.9% of the siRNA/MSN-based nanocarrier. One of the main reasons for the higher cell uptake of MSN-based nanocarrier made in this research can be considered as the presence of positive surface charge provided by PEI and guanidine. This positive charge acts as a bridge between nanoparticles and the negatively charged cell surface, promoting endocytosis. This positive charge also works inside the cell to facilitate endosomal escape according to the proton sponge theory. Another reason that justifies the high delivery of nanocarrier to breast cancer cells is the use of folate ligand. Folate receptor is overexpressed on the surface of cancer cells, which can be considered as a distinguishing factor in studies. Also, folic acid ligand has a low price and is stable in a vast temperature range, making the use of this molecule attractive for the researchers. However, its best feature is to escalation the rate of cellular uptake, which in addition to passive transfer of nanoparticles, it also causes receptor-mediated endocytosis and increases the yield of transfection^56–58^.

Figure 7B displays the CLSM images of the siRNA@[FA-PEGylated/PEI@GuIL@KIT-6] NPs incubated with MDA-MB-231 cells at 2 and 6 h explain the localization and endosomal escape promoted by these NPs. A brighter orange fluorescence signal after 2 h demonstrated that the MDA-MB-231 cells quickly ingested the siRNA@[FA-PEGylated/PEI@GuIL@KIT-6] NPs and encapsulated it into the endosomes. After 6 h of incubation, the red fluorescence gradually faded and was detected, indicating the rupture of the endosome and the escape of siRNA@[FA-PEGylated/PEI@GuIL@KIT-6] NPs from the endlysosome. This was attributed to the charge reversal of the siRNA@[FA-PEGylated/PEI@GuIL@KIT-6] NPs, which was done to facilitate cell uptake and the proton sponge effect at the endosome.

The decline in mRNA levels of EGFR1 mRNA, confirms that all steps such as cellular uptake, endosomal escape, and siRNA function have occurred appropriately. Therefore, it can be said the process of gene delivery in vitro is acceptable and in vivo steps can be taken for this gene therapy system. The PEI/siRNA complexes have been employed for non-viral gene delivery on their own, but their cytotoxicity has made in vivo applications problematic^59^. The siRNA@[FA-PEGylated/PEI@GuIL@KIT-6] is biocompatible, also it has a high affinity for encapsulation and facilitate delivery of the payload, as stated before.

## Conclusions

The fabrication of siRNA nanocarrier takes into account many factors, including those related to the purpose of transport as well as factors related to the cargo. In the present study, unique KIT-6 nanoparticles were designed by considering the most involved factors in siRNA loading into carrier nanoparticles, including the net surface charge suitable for maximum cell uptake, the proportionate amount of siRNA for cellular uptake and lack of dose-related off-target. A siRNA@[FA-PEGylated/PEI@GuIL@KIT-6] complex was synthesized to work successfully at the end of its self-efficacy pathway—the EGFR1 gene knockdown.

One of the main problems in treatment, especially targeted gene therapy, is the lysosomal digestion of a drug that has successfully passed all the barriers to cytosol. In this study, in the design of the multifunctionalized KIT-6, guanidine was used optimally to be used both in the functionalization of nanoparticles to absorb siRNA in pores of MSN and to create a buffering property of the compound for endosomal escape due to the proton sponge effect hypothesis.

Finally, manipulations such as functionalization with PEG, can have a dramatic effect, especially in in vitro cellular uptake and EGFR1 gene knockdown. Also, co-treatment of carboplatin and siRNA@[FA-PEGylated/PEI@GuIL@KIT-6] complex exhibited a remarkable cytotoxic effect in comparison to carboplatin single treatment. Study in this dimension promises to achieve an optimal and stable functionalized nanoparticle for gene therapy or co-therapy of various diseases by different siRNA sequences.

## Materials and methods

### Materials

Tetraethyl orthosilicate (TEOS), branched PEI (MW = 25 kDa), Pluronic P123 and n-butanol (n-BuOH), 3-aminopropyltriethoxysilane (APTES), and 3-chloropropyltriethoxysilane (CPTES) were acquired from Sigma Aldrich. Folic acid-PEG-acid (Folate-PEG-NH2) and trimethoxysilylpropyl modified polyethyleneimine (PEI-silane, Mw = 1500–1800), 50% in isopropanol were purchased from Biopharma PEG and Gelest companies, respectively. Scrambled siRNA (scRNA) with the sequence 50-UUC UCC GAA CGU GUC ACG UTT-30, fluorescently labeled FAM scRNA (modified at the 3’ end of sense strand), siRNA that targets EGFR1 with four distinct sequences, siR1: 50-GCG UCC GCA AGU GUA AGA ATT-30, siR2: 50-UCC ACA GGA ACU GGA UAU UTT-30, siR3: 50-CUC CAU AAA UGC UAC GAA UTT-30, siR4: 50-CCG AAA GCC AAC AAG GAA ATT-30, and positive control against GAPDH, pcRNA: 50-UGA CCU CAA CUA CAU GGU UTT-30 were synthesized by Gene Pharma Co. Ltd. (Shanghai, China). MSN-based nanocarrier was characterized by transmission electron microscopy (TEM) (Hitachi H-7650, Japan) at 80 kV, and X-Ray diffraction (XRD) (Philips X’pert 1710 diffractometer using Cu Kα (α = 1.54056 Å) in Bragg–Brentano geometry (θ-2θ)). The Fourier transform infrared spectroscopy (FTIR) was performed by a Nicolet FTIR Magna 550 spectrophotometer (USA) in the region of 4000–400 cm^−1^. Using dynamic light scattering (DLS) (Nano-ZS 90, Malvern Instrument, United Kingdom), the size distribution of nanoparticles was measured at 25 ℃. NPs surface charge resultants (Zeta Potential) were measured in folded capillary cells using Nano sizer (Zeta sizer Nano ZS90, Malvern Instruments Ltd., Malvern, UK). Cells were incubated in the bio-incubator (INCOmed246, Memmert, Germany) and all cell culture procedures were performed in a laminar hood (Aryateb. Iran). Quantitative relative qPCR was analyzed by ABI 7500 thermal cycler (Applied Biosystmes, Invitrogen, USA).

### Synthesis of nanocarrier

First step: KIT-6 was synthesized based on the procedure reported previously with a slight modification^60^ using Pluronic P123 triblock copolymer and n-BuOH as surfactant and co-surfactant. Briefly, P123 (4.0 g) was dissolved in deionized water (144 g) and HCl (35% wt, 7.9 g) afterward stirring at 35 °C, then n-BuOH (4.0 g) was added at once. TEOS and CPTES was added to this resulted clear solution and the mixture was stirred vigorously for 24 h at 35 °C. Then the mixture was aged at 100 °C for 24 h under constant conditions. Without additional washing, the white precipitate was filtered and dried under vacuum at 100 °C overnight. Then the template was removed via EtOH/HCl washing, the residual solid was dried under vacuum at room temperature to give the chloropropyl functionalized KIT-6 [Cl@KIT-6 NPs]. A suspension of [Cl-KIT-6 NPs] (0.5 g) with of guanidine, HCl (2.0 mmol) and diisopropyl ethylamine (0.36 mL, 2.1 mmol) in DMF (5 mL) was was reacted overnight. After centrifugation, the obtained fine powder was washed with EtOH and dried under vacuum for 20 h to give guanidinium IL immobilized KIT-6 [GuIL@KIT-6].

Second step: FA-PEG600–silane was prepared via the reaction between FA-PEG600-COOH (180 mg) and APTES (36 mg) reagents EDC and NHS as activation agents at room temperature for 12 h. The resulting waxy residue was washed 3 times, consequently recrystallization was occurred from dried EtOH at − 5 °C and the white waxen FA-PEG600–silane was filtered and dried at 25 °C in vacuum.

Third step: while the solution of prepared GuIL@KIT-6 (25 mg) in EtOH/H2O (30 mL,1:2) and HCl (pH = 4) was stirred vigorously, a solution of FA-PEG600–silane (0.05 g) in EtOH (30 mL) and PEI-silane (0.05 g) were added in drop-wise manner and vigorously stirred for 24 h. Afterward, the solution was filtered off and washed entirely. Eventually, the white residue was dried at 100 °C in vacuum for 12 h to attain FA-PEGylated and PEI modified GuIL@KIT-6 [FA-PEGylated/PEI@GuIL@KIT-6].

### Gel retardation assay

Gel electrophoresis was done to evaluate the binding ability of the siRNA to MSN. The buffer solutions of siRNA were mixed with [FA-PEGylated/PEI@GuIL@KIT-6] in different ratios (1:0, 1:1, 1:2, 1:6, 1:12, 1:25, 1:35 and 1:50) and incubated for 24 h at 4 °C (time and temperature was optimized in advance). A 2 μL of the mixtures was mixed with staining buffer (1 μL) and subjected to 2.5% agarose gel with Tris/acetate/EDTA (TAE) running buffer at 100 V for 45 min. RNA bands were visualized under UV light using a gel documentation system and photographed with a Bio-Rad imaging system^61^.

### Optimization of the siRNA loading

Modifying previously published Li et al.^21^the siRNA-loaded onto the [FA-PEGylated/PEI@GuIL@KIT-6] nanocarrier (called siRNA@[FA-PEGylated/PEI@GuIL@KIT-6]) was prepared. The effect of two factors including the temperature and loading time for ratio of 1:35 of siRNA/MSN was examined to optimize the siRNA loading in [FA-PEGylated/PEI@GuIL@KIT-6]. The ratio 1:35 of siRNA/MSN was achieved from gel retardation assay which the whole complex with neutral net charge was formed. An aqueous solution of siRNA and [FA-PEGylated/PEI@GuIL@KIT-6] were added into a 2 mL centrifuge tube. The mixture was vortexed for 40 s and dispersed by continues shaking with 280 rpm 1 h at 4 °C, 1 h at 25 °C, 24 h at 4 °C and 24 h at 25 °C. The loading efficiency of KIT-6-based nanoporous with various pore sizes also was evaluated. The solid residual was centrifuged at 20,000*g* for 15 min to separate the siRNA@[FA-PEGylated/PEI@GuIL@KIT-6]. The siRNA amount in supernatant was measured using Take3 Micro-volume plates, Epoch Microplate Spectrophotometer (BioTek).

### siRNA release assay

In TE buffer, the release of siRNA was evaluated according to Liu et al. procedure^23^. In RNase-free Eppendorf 2.0 mL tubes, siRNA@[FA-PEGylated/PEI@GuIL@KIT-6] was added to 200 µl TE buffer and maintained at 37 °C with horizontal shaking (120 rpm/min). The tubes were centrifuged (12,000 rpm/min, for 30 min) at 4 °C in specified intervals. The siRNA@[FA-PEGylated/PEI@GuIL@KIT-6] deposits were re-dispersed in new TE buffer to re-incubate in 37 °C, and the NPs supernatants were quantified at 260 nm with Take3 Micro-volume plates, Epoch Microplate Spectrophotometer (BioTek).

### RNase assay

To study the protection effect of the siRNA by [FA-PEGylated/PEI@GuIL@KIT-6] against RNase, siRNA@[FA-PEGylated/PEI@GuIL@KIT-6] after washing with PBS was added to the RNase in solution. The solution was incubated for 1 h with 0.25% RNase A (Takara) and were loaded on a 2.5% agarose gel and run at 90 V for 40 min. To release the bound siRNA from noncomplex, the mixtures were treated with 0.6 U/mg of heparin per mg siRNA in DEPC-treated water before gel electrophoresis.

### Cell culture

The Triple negative human breast cancer cell line and human breast normal cell line, MDA-MB-231 and MCF-10A, were purchased from National Cell Bank of Iran (NCBI) and cultured in RPMI 1640 (Gibco) in an incubator at 5% CO2, 37 °C and humidified atmosphere. RPMI 1640 medium was supplemented with 10% heat-inactivated fetal bovine serum (FBS, Biosera) 100 mg/mL streptomycin and 100 U/mL penicillin.

### Cell survival assay

The anti-proliferation activity of siRNA@[FA-PEGylated/PEI@GuIL@KIT-6] and carboplatin was determined by MTT [3-(4, 5- dimethylthiazol-2-yl)-2, 5-diphenyl tetrazolium bromide] assay against MDA-MB-231 cell line. Also, the cytocompatibility of [FA-PEGylated/PEI@GuIL@KIT-6] was determined against MDA-MB-231 and MCF-10A. In brief, approximately 1 × 10^4^ cells per well were seeded in 96-well plates in 190 µl of RPMI 1640 overnight. The cells were transfected with serial dilutions of compounds that was diluted in culture medium without serum and antibiotics. Non-transfected cells were considered as negative controls. After 24 and 48 h of incubation at 37 °C, 5% CO_2_, and 90% humidity, the medium was removed and 200 μL phenol red-free medium containing MTT (1 mg/mL) was added to each well and the plates were incubated for another 4 h. Finally, formazan crystals in each well were dissolved with 100 μL of DMSO and the absorbance at 570 nm was measured using a microplate reader (Epoch, BioTek). The experiments were conducted 3 times.

### Flow cytometry analysis of the apoptotic cells with AnnexinV-PE/7-AAD double staining

By using flow cytometry, the apoptosis rate of MDA-MB-231 cells was identified and measured^62^. This approach was used to discriminate between live cells (annexin V-PE^−^/7-AAD^−^), early apoptotic cells (Annexin V^-^PE^+^/7-AAD^−^), late apoptotic or secondary apoptotic cells (Annexin V^−^PE^+^/7-AAD^+^), and necrotic cells (Annexin V^−^PE^−^/7-AAD^+^). Briefly, 3 × 10^5^ cells per well of MDA-MB-231 in 6-well plate were transfected by siRNA@[FA-PEGylated/PEI@GuIL@KIT-6] and Carboplatin. After 24 h treatment treated and untreated cells as negative control were collected, harvested, and incubated with Annexin V and 7-AAD for 15 min at 20 °C in the dark. Flow cytometry (Sysmex cyflow space, Japan) was used to examine the cells.

### Cellular uptake flow cytometry analysis

For cellular uptake of siRNA, 3 × 10^5^ cells per well of MDA-MB-231 were seeded in a 6-well plate overnight. The culture medium was replaced by fresh low serum medium containing the complex of FAM-labeled siRNA loaded in [FA-PEGylated/PEI@GuIL@KIT-6]. After incubating for 2 and 4 h, the cells were washed with PBS solution 3 times, trypsinized, harvested and re-suspended in PBS. For quantification of the intracellular uptake of FAM labeled siRNA-MSN, MDA-MB-231 cells were analyzed using the flow cytometer (Sysmex cyflow space, Japan) (FL1, 525 ± 20 nm) within 1 h.

### Localization and endolysosomal escape

Using CLSM, the siRNA trafficking inside cells was evaluated. In RPMI-1640 containing 10% FBS, MDA-MB-231 cells were seeded onto a coverslip in 35 mm culture plate, and the cells were left to adhere for 24 h. In a media without serum, the cells were treated by FAM-labeled siRNA@[FA-PEGylated/PEI@GuIL@KIT-6]. The cells were incubated for 2 and 4 h before being washed twice with PBS, fixed for 1 h in 1.5 mL of deep red lysotracker (100 nM in RPMI) for 1 h. The lysosomal dye was then discarded and the cells were wash with PBS, fixed with 4% formaldehyde for 5 min, and stained for 10 min with DAPI (200 ng/mL). The cells were then examined using a CLSM (Zeiss, Germany).

### mRNA extraction, cDNA synthesis and qPCR

RNA purification was performed according to the previously published method (Haghighat et al. 2017). In summary, cells were collected using the total RNA purification QIAGEN RNeasy Kit and the rest of the steps were performed according to the manufacturer’s instruction. The concentration of purified mRNA was measured using a NanoDrop Spectrophotometer (Biotek, Japan). Yields were calculated and using reverse transcription & cDNA synthesis for qPCR kit (QIAGEN), 100 ng of mRNA was reverse -transcribed to complementary DNA (cDNA). The first-strand cDNA was synthesized by reverse transcriptase (M-MuLV). Then quantitative relative qPCR was performed by intercalating SYBR Green dye in a reaction volume of 20 μL, according to the manufacturer’s instructions (Ampliqon, Denmark). In a few words, 10 μL of master mix, of primers (4 μL) (10 pMol), template cDNA (2 μL) (1.5 μg/μL) and ddH2O (4 μL) were added to each well. Then, the fast PCR was performed for 40 cycles of the following program: (i) activation at 95 °C for 3 min, (ii) denaturation at 95 °C for 15 s and (iii) annealing and extension at 60 °C for 1 min. The qPCR result was obtained by the fluorescent intensity emitted due to amplification of a piece (78 nt) of the Homo sapiens EGFR1 (NM_005228.5) mRNA and GAPDH (NM_001256799) mRNA, using exon junction forward and reverse specific primers, FEGFR: 5′-AGG TGG TCC TTG GGA ATT TG-3′, REGFR: 5′-CCT CCT GGA TGG TCT TTA AGA AG-3′, FGAPDH: 5′-CTG GGC TAC ACT GAG CAC C-3′ and RGAPDH: 5′-AAG TGG TCG TTG AGG GCA ATG-3′. Raw data obtained from amplification was normalized to GAPDH as the reference gene and were represented as Cq values.

All qPCR reactions were run in triplicate on an ABI 7500 Real-time PCR system. To investigate the EGFR1 expression and the precise amount of gene knockdown, the comparative 2^−ΔΔCT^ (2^−ΔΔCq^) method was performed. The C_q_ with 0.05 threshold was used to determine the knockdown level in the treated and untreated cells 24 and 48 h after incubating with siRNA. The gene knockdown level was then calculated as published previously^63^. The results were represented as the ratio of target to reference gene, using the following formula: ΔΔCq = ΔCq (Treated)—ΔCq (Control), where ΔCq for treated cells is ΔCq_treated_ = Cq (EGFR1 in treated cells)—Cq (GAPDH in treated cells) and for untreated cells is ΔCq_control_ = Cq (EGFR1in untreated cells)—Cq (GAPDH in untreated cells). Therefore, the amount of knockdown is calculated as a fraction of the amount of gene expression level.

### Statistics

Data were analyzed using GraphPad Prism version 8.4.2 (GraphPad Software, Inc., San Diego, CA). Statistical significance was assessed by one-way ANOVA and Student’s *t* test. P < 0.05 was considered as the significance criterion. The date was represented as a bar graph (mean and standard deviation).

## Supplementary Information

Supplementary Figure 1.

## Data Availability

The data that support the findings of this study are available from the corresponding author upon reasonable request.
