# Supplementary material for: Overcoming the non-kinetic activity of EGFR1 using multi-functionalized mesoporous silica nanocarrier for in vitro delivery of siRNA
Source: Sci Rep. 2022 Oct 14;12:17208. doi: 10.1038/s41598-022-21601-w (PMC9568566; doi:10.1038/s41598-022-21601-w)
Supplement: Supplementary file 1 — Supplementary Figure 1. [file 41598_2022_21601_MOESM1_ESM.docx]

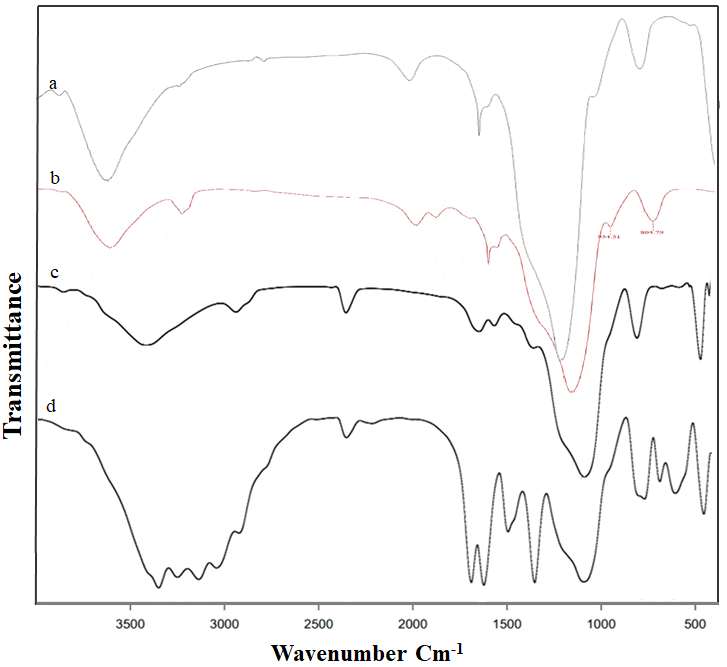


**Supplementary Information 1.** FTIR spectra of (a): [KIT-6], (b): [PEGylated/PEI@GuIL@KIT-6], (c): [GuIL@KIT-6], (d): [FA-PEGylated/PEI@GuIL@KIT-6].
